# Primary Outcomes of Accelerated Epithelium-Off Corneal Cross-Linking in Progressive Keratoconus in Children: A 1-Year Prospective Study

**DOI:** 10.1155/2017/1923161

**Published:** 2017-12-31

**Authors:** Sherif A. Eissa, Nashwa Badr Eldin, Ashraf Ahmed Nossair, Wael Ahmed Ewais

**Affiliations:** Department of Ophthalmology, Kasr Al Ainy Hospital, Faculty of Medicine, Cairo University, Kasr Alainy, Cairo, Egypt

## Abstract

**Purpose:**

To evaluate corneal transparency following accelerated collagen cross-linking (ACXL) in pediatric keratoconus.

**Design:**

A prospective interventional case series.

**Methods:**

This study included 47 eyes (25 patients), aged 9–14 years, with documented progressive keratoconus. After applying 0.1% riboflavin drops, ACXL was performed. Assessment included corrected distance visual acuity (CDVA), uncorrected visual acuity (UCVA), corneal haze, and corneal densitometry in grayscale units (GSU).

**Result:**

The mean baseline and corneal densitometry peaked at 3 months post-ACXL while central and posterior densitometry showed a statistically significant increase (*P* < 0.05) and peaked at 8 months postoperatively. By 12 months, densitometry in all corneal layers (*P* ≥ 0.99) and concentric zones (*P* ≥ 0.97) reached near baseline values. Slit-lamp graded haze peaked at 1 month to 1.82 ± 0.65 (*P* < 0.05) and declined to near baseline at 12 months (0.39 ± 0.58). There was a statistically significant increase in the mean UCVA and CDVA at 12 months.

**Conclusion:**

Total and anterior corneal densitometry peaked after 3 months, while central and posterior densitometry peaked after 8 months. Maximum haze was at 1 month post-ACXL. All corneal layers, concentric zone densitometry and haze reached near baseline values after 1 year. Scheimpflug densitometry showed weak correlation with CDVA over the 12-month follow-up period (*r* = −0.193).

## 1. Introduction

Visual rehabilitation after CXL, especially in children, may be prolonged up to 12 months postoperatively, with variable courses and predisposing factors [1].

Wollensak et al. first introduced CXL for the treatment of progressive keratoconus in 2003. It aims at halting the progression of keratoconus and deferring the need for keratoplasty [2]. Standard CXL with 30 minutes of exposure to UV irradiation of 3 mW/cm^2^ as described in the Dresden protocol proved to increase its biomechanical strength in the anterior 200 *μ*m of the corneal stroma [3].

Intracorneal ring segment (ICRS) implantation in pediatric keratoconus management is the second option. However, ICRS implantation is not preferred in children owing to the aggressive nature of pediatric keratoconus and the tendency of eye rubbing in children [4].

On the other hand, corneal CXL proved to succeed in halting the progression of keratoconus in pediatric patients, in addition to improvements in visual and topographic measurements [5]. However, long-term follow-up is crucial to detect any signs of progression, as reported by Godefrooij et al., who described disease progression in 22% of the pediatric keratoconus eyes treated with standard epithelium-off CXL [6].

Secondary to backward light scattering, the developed corneal haze following CXL can be detected clinically and graded subjectively on slit-lamp biomicroscopy and evaluated quantitatively using Scheimpflug densitometry [7]. The increased collagen fiber diameter [8], keratocyte apoptosis, and subsequent transformation into myofibroblasts [9] are the reported corneal changes on transmission electron microscopy that interpret the reduction in corneal transparency noted post-CXL.

Kamaev et al. found higher densitometry values in anterior stroma compared to middle and posterior stroma, as well as the central 6 mm zone compared to the more peripheral zones. The decline in riboflavin concentration, intensity of ultraviolet light, and oxygen concentration in deeper stromal layers explains the higher anterior stromal values [10], whereas the Gaussian beam profile of the conventional UVA-irradiation device with increased irradiation intensity at the center and gradual decline towards the periphery explains the more central haze distribution [11].

In this study, we adopted accelerated cross-linking (ACXL) treatment in pediatric keratoconus because of its biomechanical strengthening effects on the corneal tissue, commensurate to the standard protocol [12], and the previously reported improvement in UCVA and CDVA post-ACXL [13]. We aimed in the current study to evaluate corneal transparency and visual acuity following ACXL in pediatric keratoconus in a highly populated region with rapidly progressive keratoconus, south KSA (Kingdom of Saudi Arabia).

## 2. Subjects and Methods

Forty-seven eyes of 25 patients with progressive keratoconus, aged 9–14 years, were involved in this prospective study at Magrabi Eye Hospital (M. E. H.). The ethical committee of (M. E. H.) in Aseer province approved the study as well as the IRB, which prospectively approved the design and implementation of the procedure on children. Comprehensive discussion with parents was undertaken before surgery, explaining to them the details of the procedure and its benefits and complications. An informed written consent was obtained from all parents. One surgeon performed all the surgeries (S. E.).

Inclusion criteria were the patients' age ≤ 14 years, corneal thickness at the thinnest location of 400 *μ*m or more, and clear cornea with documented diagnosis of progressive keratoconus. Exclusion criteria were a corneal thickness less than 400 *μ*m, history of viral keratitis, concurrent corneal infection, the presence of corneal stress lines or corneal scarring, K max over 53 D, and endothelial cell density less than 2500 cells/mm^2^.

The frequency of preoperative visits was every 3 months for keratoconus suspects with one or more of the following criteria: positive family history of keratoconus, incomplete corneal Fleischer ring, high astigmatic error, frequent change of spectacle prescription, and headache.

In the current study, abnormal pattern on the sagittal map (crab claw pattern or asymmetric bowtie with skewed radial axes), plus one of the following signs, corneal thickness less than 470 microns or K max above 47 D, were essential for the early diagnosis of keratoconus by Scheimpflug corneal tomography (Pentacam, Oculus Optikgerte GmbH, Wetzlar, Germany) [14, 15].

We considered keratoconus to be progressive when K max increased by ≥1.0 D or pachymetry of the thinnest location decreased by ≥5%, on two consecutive corneal tomographic examinations [16] taking into consideration the inversely correlated severity of keratoconus with age [17] and highly significant risk of performing keratoplasty with age< 18 years at presentation [18].

Diagnosis of amblyopia in any keratoconus case was confirmed preoperatively, if CDVA did not improve with rigid gas permeable contact lens trial, in the absence of another ocular pathology. The importance and need of postoperative spectacle prescription and patching of the healthy eye were explained to the parents.

## 3. Surgical Technique

Under general anesthesia, the same surgeon (S. E.) performed all procedures. Following epithelial peeling and removal from 9 mm treatment zone, riboflavin 0.1% (Vibex Rapid, Avedro Inc., Waltham, MA, USA) containing hydroxypropyl methylcellulose (HPMC) was applied for 15 minutes. The cornea was then exposed to ultraviolet light of non-Gaussian, uniform top hat beam profile and wavelength 365 nm (Kxl, Avedro Inc., Waltham, MA, USA), at 18 mW/cm^2^ for 5 minutes, to deliver total energy of 5.4 J/cm^2^ [19]. Permeation of riboflavin through the cornea was confirmed using a portable slit-lamp (PSL) (Reichert, Depew, NY, USA), before exposure to UV light. Riboflavin was applied to the corneal surface every 90 seconds, to prevent corneal dryness during the 5-minute exposure to UV light. A polymethyl methacrylate limbal guard was used to protect the limbal stem cells during exposure to the 9 mm-sized beam. At the end of the procedure, a silicone hydrogel-bandage contact lens (ACUVUE®, Johnson & Johnson Vision Care Inc., FL, USA) was applied. Postoperatively, we used topical moxifloxacin hydrochloride 0.5% (Vigamox®; Alcon, TX, USA), dexamethasone 0.1%, and tobramycin 0.3% (Tobradex®; Alcon, TX, USA) hourly on the day of surgery. After 1 day, the frequency was reduced to 6/day for steroid and 4/day for antibiotic drops for 14 days. Fluorometholone 0.1% replaced Tobradex for 2 more weeks.

In our study, high-fluency CXL was based on the Bunsen-Roscoe law of photochemical reciprocity, which states that the same photochemical effect can be achieved with a reduced irradiation time, provided that the total energy level is kept constant through a corresponding increase in irradiation power [19].

Follow-up visits were at 1 week, 1, 3, 8, and 12 months following ACXL. Assessment included spectacle corrected and uncorrected distance visual acuity in decimal, intraocular pressure (IOP), fundus examination, corneal haze grading by slit-lamp, endothelial cell count (ECC) (Konan Medical, Torrance, CA, USA), the steepest keratometry (K max), corneal thickness, and corneal densitometry (Pentacam, Oculus Optikgerte GmbH, Wetzlar, Germany) in standardized grayscale units(GSU). Clinical corneal haze, corneal densitometry, and CDVA were compared to preoperative values for any statistical significance. All patients (47 eyes) attended the scheduled visits up to 1 year postoperatively.

## 4. Assessment of Corneal Transparency

We clinically evaluated corneal transparency as a primary variable following ACXL. Secondary variables included Scheimpflug densitometry and visual acuity. The corneal haze grading system adopted in this study was described by Greenstein et al. [1] and was modified by the author for a simpler application, with less subjective variability. Grade 0+ stands for the clear cornea, grade 1+ for focal minimal stromal cloudiness, grade 2+ for diffuse stromal cloudiness, and grade 3+ for diffuse dense stromal cloudiness obscuring iris details. Clinically, slit-lamp grading of corneal transparency was monitored every visit and recorded based on the aforementioned grading system.

**Figure 1:**
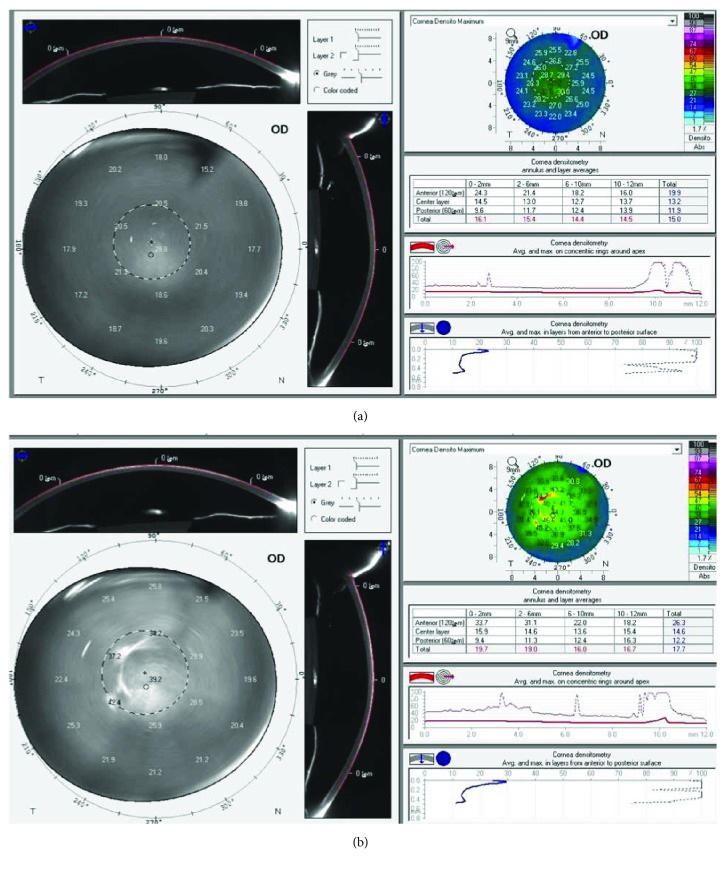
Accelerated collagen cross-linking in progressive pediatric keratoconus. A demonstration of corneal densitometry measurement using Scheimpflug tomography, before accelerated cross-linking (ACXL) (a) and after ACXL (b) in pediatric keratoconus.

In Scheimpflug-based corneal densitometry (Pentacam, Oculus Optikgerte GmbH, Wetzlar, Germany), the backward light scatter was measured for anterior 120 *μ*m of stroma, posterior 60 *μ*m, and central stroma in between, in three concentric zones, 0.0 to 2.0 mm, 2.0 to 6.0 mm, and 6.0 to 10.0 mm (Figure 1).

## 5. Statistical Analysis

SPSS 15 software (SPSS Inc., Chicago, Illinois, USA) was used for statistical analysis. All normally distributed continuous data were presented as means and standard deviations. Ordinal data were expressed as median (range). Repeated measure ANOVA was used for data analysis, followed by a post hoc test. A *P* value of <0.05 was considered statistically significant. A sample size of 47, giving an alpha value of 0.05 with a power of 90%, was calculated using the Lerman method.

## 6. Results

**Figure 2:**
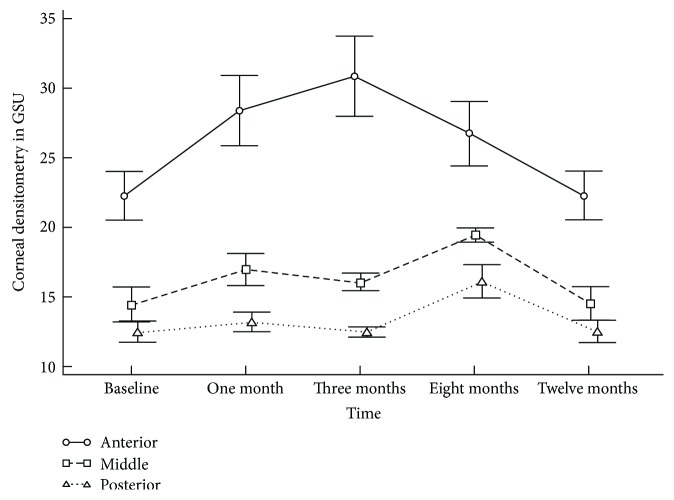
Accelerated collagen cross-linking in progressive pediatric keratoconus. Corneal densitometry—in grayscale units (GSU)—in the three corneal layers throughout follow-up time points. Data are presented as mean; bars are standard deviation (SD).

**Figure 3:**
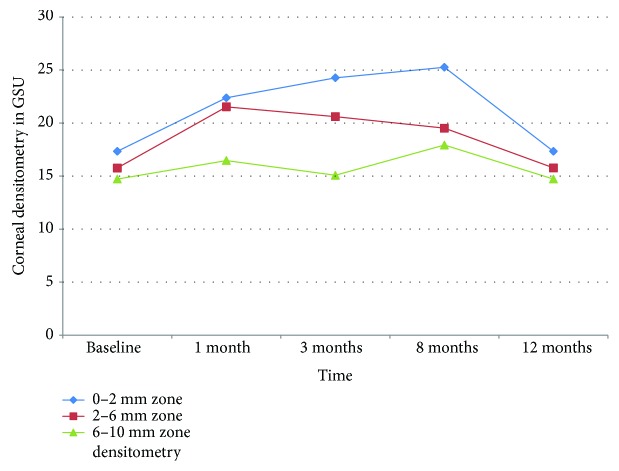
Accelerated collagen cross-linking in progressive pediatric keratoconus. Densitometry—in GSU—of three concentric zones at different time points.

The Scheimpflug corneal densitometry in the anterior, middle, and posterior stromal layers had increased during the postoperative period (Table 1). The preoperative mean densitometry of 18.34 ± 0.72 GSU peaked to 22.25 ± 0.35 GSU at 3 months (*P* < 0.05). The densitometry values peaked at 3 months in anterior stromal layer (30.92 ± 2.86 GSU), while peak values of densitometry in central (19.50 ± 0.49 GSU) and posterior (16.15 ± 1.18 GSU) stroma were at 8 months postoperatively (Figure 2). At 1 year, densitometry values reached near baseline values, with approximation in all layers. A statistically significant increase in the mean densitometry in all three zones of the entire depth of the cornea persisted until 8 months postoperatively. All measurements showed higher mean densitometry values in the 0.0 to 2.0 mm zone than in the 2.0 to 6.0 and 6.0 to 10.0 mm zones. However, 1-month densitometry at 0.0 to 2.0 mm showed close measures to the 2.0 to 6.0 mm zones (Figure 3). Scheimpflug densitometry showed weak correlation with CDVA over the 12-month follow-up period (*r* = −0.193) (Table 2).

**Figure 4:**
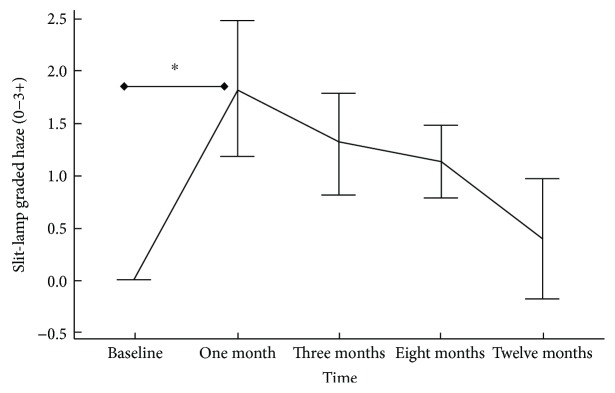
Accelerated collagen cross-linking in progressive pediatric keratoconus. Time course of ACXL-associated corneal haze, graded by slit-lamp biomicroscopy (0–3+). Data are presented as mean; bars are SD. ^∗^*P* value < 0.05.

Slit-lamp grading of corneal transparency showed a peak in mean clinical haze at 1 month (1.82 ± 0.65) postoperatively (*P* < 0.05) that declined thereafter to (0.39 ± 0.58) at 1 year (Figure 4).

**Figure 5:**
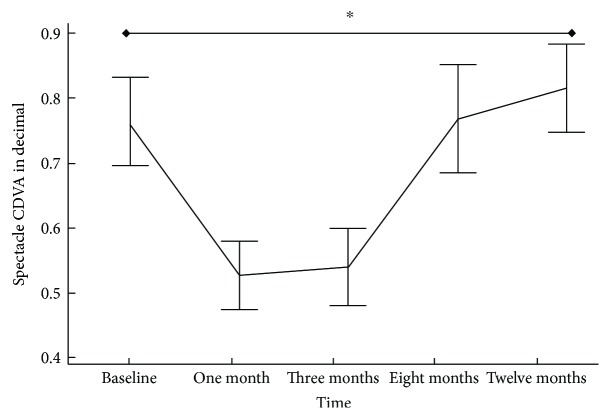
Accelerated collagen cross-linking in progressive pediatric keratoconus. Spectacle-corrected distance visual acuity (CDVA)—in decimal—after ACXL at different time points. Data are presented as mean; bars are SD. ^∗^*P* value < 0.05.

The mean preoperative UCVA and CDVA were 0.32 ± 0.05 and 0.76 ± 0.06, respectively, that at the end of 12 months showed a statistically significant improvement (Table 3). The reduction in mean CDVA (Figure 5) was statistically significant at 1 month (*P* < 0.05) and 3 months (*P* < 0.05). Additionally, the three cases encountered with anisometropic amblyopia and managed by postoperative spectacle prescription and patching of the sound eye gained 1 line of CDVA at 12 months post-ACXL. UCVA showed moderate correlation with HOAs (*r* = 0.436) 12 months postoperatively.

The pachymetry map showed a nonstatistically significant decrease in preoperative thinnest location by a mean of 0.32 ± 0.85 D (Table 3). Baseline steepest K (47.19 ± 1.62 D) showed a nonstatistically significant decrease (46.87 ± 0.77 D) at 12 months postoperatively (Table 3).

Regarding wavefront high-order aberrations (HOAs) in RMS, mean preoperative value of 6.86 ± 3.17 that decreased to 5.79 ± 2.98, 5.66 ± 2.81, 5.62 ± 2.80, and 5.62 ± 2.80 at postoperative 1, 3, 8, and 12 months, respectively.

Corneal densitometry showed only moderate correlation (*r* = 0.308) with HOAs preoperatively. However, postoperatively, densitometry showed weak correlation with UCVA (*r* = −0.192), HOAs (*r* = −0.147), central corneal thickness (*r* = −0.012), and K max (*r* = 0.006) readings after 1 year.

In no case did epithelial defect persists for more than a week, and mean healing time was 4 ± 1.15 days. We did not encounter complications like delayed reepithelialization, endothelial decompensation, cataract, uveitis, or corneal infection throughout the 12-month follow-up. Moreover, baseline ECC of 2945.2 ± 138.5 cells/mm^2^ showed a nonstatistically significant reduction to 2838.9 ± 134.07 at 12 months post-ACXL (*P* > 0.05).

## 7. Discussion

We applied ACXL treatment in pediatric keratoconus in our study, encouraged by its positive results, namely, its biomechanical stiffening effect on corneal tissue [12] and the statistically significant improvement in UCVA and CDVA [13].

The superior biomechanical stability of the epithelium-off CXL, 320% versus 64% in epithelium-on CXL, [3] may be explained by the blockage of adequate penetration of riboflavin into the corneal stroma through intact epithelium [20] and may explain the inferior efficacy of the epithelium-on CXL in terms of its ability to halt keratoconus progression in children, with 0.70 of the efficacy of epithelium-off CXL [21].

Despite offering less intraoperative time consumption and less postoperative pain, we did not use the epithelium-on technique, owing to its major limitations that include limited UVA stromal penetration, [22] the use of the same power of UVA as for standard treatment (3 mW/cm^2^) [23], and being 70% less effective in terms of biomechanical strength than standard epithelium-off CXL [24].

Applying the Dresden protocol, Zotta et al. [25], Arora et al. [26], McAnena and O'Keefe [27], and Uçakhan et al. [5] declared the use of CXL for children with progressive keratoconus to be efficient in improving CDVA and UCVA. The latter study reported significant improvements in topographic, aberrometric, and elevation indices after 6 months postoperatively, in 40 pediatric eyes, with a follow-up period of 4 years.

In our study, we did not encounter keratoconus progression, unlike Caporossi et al. who reported a clinically significant deterioration in all parameters in 50% of pediatric keratoconus patients after 1 year of transepithelial CXL [28].

Few studies reported the use of ACXL in pediatric keratoconus, like Shetty et al. who documented an improvement in the mean UCVA, CDVA, and keratometry at 2 years post-ACXL [29]. Ozgurhan et al. enrolled 44 eyes of 38 children with progressive keratoconus, who underwent a 4-minute ACXL, followed by a significant CDVA improvement and decreased steep keratometry, and keratoconus progression was halted [30], which agrees with our results. However, corneal haze was analyzed neither clinically nor with Pentacam densitometry.

Several studies analyzed the corneal densitometry measures following standard CXL in adult keratoconus patients. In agreement with our study, Greenstein et al. [1], Alnawaiseh et al. [31], Gutiérrez et al. [32], and Pircher et al. [7] reported a peak of corneal densitometry in the first months after standard CXL that returned to preoperative values approximately 1 year after CXL.

The study conducted by Gutiérrez et al. [32] reported detectable densitometry changes in the absence of concomitant clinical haze beyond grade 1+. However, they did not report densitometry changes on the basis of separate layers and concentric zones. Greenstein et al. [1] found the absolute measurement of CXL-associated corneal haze measured by densitometry at 12 months to correlate significantly with CDVA (*r* = −0.71). However, in our study, the changes in densitometry were not correlated with the change in CDVA from baseline to 12 months.

In contrast with the results of our study, Shen et al. [33] reported a dramatic decrease in the densitometry values over 12 months following accelerated (320 seconds) transepithelial CXL. However, the latter retrospective cohort study enrolled only 17 keratoconus eyes, of older age group (22.4–31.4 years), exposed to 45 mW/cm^2^ (total energy: 7.3 J/cm^2^) irradiance of epithelium-on ACXL. The epithelium removal in epi-off cross-linking may trigger more severe inflammatory responses with more keratocyte activation [9] and may explain the higher early postoperative densitometry values when compared to accelerated transepithelial CXL [9].

In our study, a 12-month densitometry showed a weak correlation with CDVA (*r* = −0.19). These Scheimpflug densitometry changes were benchmarked against the slit-lamp clinical grading of corneal haze (Figures 2 and 4) throughout its natural course up to 12 months. The time course of clinical corneal haze after CXL was objectively quantified; it was greatest at 1 month and was significantly decreased between 3 and 12 months. However, postoperative peaking of total and anterior corneal layer densitometry (30.92 ± 2.86 GSU) at 3 months may interpret a subclinical haze, with failure of improvement in CDVA despite decreased slit-lamp clinical haze. The synchronized trough of total, anterior densitometry, and slit-lamp haze with CDVA peak (Figures 2 and 5) at 1 year suggests that densitometry does measure clinical corneal haze.

A study by Lamy et al. [34] in 2013 assessed the effects of CXL on contrast sensitivity, visual acuity, and corneal topography as possible predictors of efficacy of CXL treatment. They reported a statistically significant improvement in contrast sensitivity from a mean of 1.53 ± 0.12 logCS to 1.69 ± 0.1 logCS (*P* < 0.001) over 24 months of follow-up. They also reported an improvement of −0.16 logarithm of the minimum angle of resolution (logMAR) and a reduction in SimK-s of −0.61 diopter. However, high-order aberrations were not investigated in this study, despite being suggested as a cause of degrading contrast sensitivity and visual acuity in keratoconus patients [35].

One of the disadvantages of our study was the short follow-up period, 1 year. Besides, we did not include in our study the assessment of contrast sensitivity and its correlation with corneal haze and CDVA following ACXL in pediatric keratoconus.

Based on our study and the study by Lamy et al., we recommend the design of future prospective randomized studies that investigate the possible correlation between corneal densitometry and contrast sensitivity after conventional and accelerated CXL.

One of the advantages of ACXL in children is the short duration of exposure to general anesthesia (GA), adding an immense benefit in reducing GA risk and may result in better cooperation when topical anesthesia is used in pediatric patients. Children with Down syndrome and keratoconus, which is a common association, would benefit from this advantage. Such children have also been observed to have cardiac and chest problems, apart from difficult visual acuity assessment, and this renders exposure to short duration of anesthesia a crucial advantage.

To our knowledge, this is the first study to evaluate the impact of ACXL in progressive pediatric keratoconus on corneal transparency, and its correlation with densitometry and CDVA. This prospective study that aimed to objectively clarify the natural course of corneal haze could convey to the ophthalmologists an interpretation of the subclinical barriers to a smooth visual rehabilitation after CXL.

The safety and efficacy of ACXL treatment in pediatric keratoconus in our study were verified by the improved CDVA, near physiological decrease in ECC, the ameliorated keratoconus progression, and resolved corneal haze by 12 months postoperatively. However, a larger prospective controlled case study with longer follow-up is required to compare corneal haze in three groups, accelerated CXL, 30-minute UV-standard epithelium-off CXL, and epithelium-on CXL.

Following CXL in children, Pentacam-based corneal densitometry values in various corneal zones and depths can add to the clinicians' adjunctive reliable tools to independently and objectively assess, verify, and report the subjective grading of clinical slit-lamp-based corneal haze, in correlation with the measured or anticipated visual acuity throughout the follow-up period.

Based on the current study and the study by Lamy et al. in 2013, larger prospective randomized studies are required to investigate the possible correlation between corneal densitometry and contrast sensitivity following both conventional and accelerated CXL.

In conclusion, total and anterior layer corneal densitometry peaked after 3 months, while central and posterior densitometry peaked after 8 months. Maximum slit-lamp graded haze was at 1-month post-ACXL. All corneal layers and concentric zone densitometry and slit-lamp graded haze reached near baseline values after 1 year. Scheimpflug densitometry showed weak correlation with CDVA over the 12 months follow-up period (*r* = −0.193).

## Figures and Tables

**Table 1:** Scheimpflug densitometry measurements in stromal layers and concentric zones over time.

	Anterior stroma	Central stroma	Posterior stroma	0–2 mm zone	2–6 mm zone	6–10 mm zone
Baseline	22.303 ± 1.772	14.503 ± 1.22	12.55 ± 0.77	17.33 ± 0.85	15.76 ± 0.733	14.72 ± 1.43
1 month	28.41 ± 2.55	17.02 ± 1.14	13.24 ± 0.68	22.38 ± 3.52	21.52 ± 0.22	16.46 ± 0.43
3 months	30.92 ± 2.86^∗^	16.07 ± 0.62^∗^	12.52 ± 0.36^∗^	24.26 ± 0.63^∗^	20.60 ± 0.50^∗^	15.07 ± 0.68^∗^
8 months	26.78 ± 2.30^∗^	19.50 ± 0.49^∗^	16.15 ± 1.18^∗^	25.26 ± 1.09^∗^	19.51 ± 0.92^∗^	17.92 ± 2.3^∗^
12 months	22.30 ± 1.77	14.50 ± 1.22	12.55 ± 0.77	17.33 ± 0.85	15.77 ± 0.72	14.72 ± 1.43

^∗^ *P* value < 0.05 is considered statistically significant.

**Table 2:** Correlations (*r* value) and significance (*P* value) between CDVA and densitometry over time.

		Corneal densitometry and CDVA
*r* value	Baseline	−0.065
*P* value		0.769
*r* value	1 month	0.362
*P* value		0.08
*r* value	3 months	0.136
*P* value		0.53
*r* value	8 months	−0.054
*P* value		0.806
*r* value	12 months	−0.193
*P* value		0.378

CDVA: corrected distance visual acuity.

**Table 3:** Changes in CDVA, UCVA, steepest K, and corneal thickness at 12 months post-ACXL.

	Preoperative	1 year postoperative	*P* value
CDVA (decimal)	0.76 ± 0.06	0.815 ± 0.05	0.013
UCVA (decimal)	0.32 ± 0.05	0.40 ± 0.05	0.002
K max (diopters)	47.19 ± 1.62	46.87 ± 0.77	0.308
Pachymetry at thinnest location (microns)	467.30 ± 3.39	460.72 ± 22.17	0.097

CDVA: corrected distance visual acuity; UCVA: uncorrected visual acuity; K: keratometry; ACXL: accelerated collagen cross-linking.
